# Diallyl disulfide suppresses epithelial-mesenchymal transition, invasion and proliferation by downregulation of LIMK1 in gastric cancer

**DOI:** 10.18632/oncotarget.7252

**Published:** 2016-02-08

**Authors:** Bo Su, Jian Su, Ying Zeng, Fang Liu, Hong Xia, Yan-Hua Ma, Zhi-Gang Zhou, Shuo Zhang, Bang-Min Yang, You-Hua Wu, Xi Zeng, Xiao-Hong Ai, Hui Ling, Hao Jiang, Qi Su

**Affiliations:** ^1^ Center for Gastric Cancer Research of Hunan Province, First Affiliated Hospital, University of South China, Hengyang, 421001 Hunan, China; ^2^ Key Laboratory of Cancer Cellular and Molecular Pathology of Hunan Provincial University, Cancer Research Institute, University of South China, Hengyang, 421001 Hunan, China; ^3^ Key Laboratory for Pharmacoproteomics of Hunan Provincial University, Institute of Pharmacy and Pharmacology, University of South China, Hengyang, 421001 Hunan, China; ^4^ Department of Pathology, Second Affiliated Hospital, University of South China, Hengyang, 421001 Hunan, China

**Keywords:** diallyl disulfide, LIMK1, gastric cancer cell epithelial-mesenchymal transition, invasion, proliferation

## Abstract

Diallyl disulfide (DADS) has been shown to have multi-targeted antitumor activities. We have previously discovered that it has a repressive effect on LIM kinase-1 (LIMK1) expression in gastric cancer MGC803 cells. This suggests that DADS may inhibit epithelial-mesenchymal transition (EMT) by downregulating LIMK1, resulting in the inhibition of invasion and growth in gastric cancer. In this study, we reveal that LIMK1 expression is correlated with tumor differentiation, invasion depth, clinical stage, lymph node metastasis, and poor prognosis. DADS downregulated the Rac1-Pak1/Rock1-LIMK1 pathway in MGC803 cells, as shown by decreased p-LIMK1 and p-cofilin1 levels, and suppressed cell migration and invasion. Knockdown and overexpression experiments performed *in vitro* demonstrated that downregulating LIMK1 with DADS resulted in restrained EMT that was coupled with decreased matrix metalloproteinase-9 (MMP-9) and increased tissue inhibitor of metalloproteinase-3 (TIMP-3) expression. In *in vitro* and *in vivo* experiments, the DADS-induced suppression of cell proliferation was enhanced and antagonized by the knockdown and overexpression of LIMK1, respectively. Similar results were observed for DADS-induced changes in the expression of vimentin, CD34, Ki-67, and E-cadherin in xenografted tumors. These results indicate that downregulation of LIMK1 by DADS could explain the inhibition of EMT, invasion and proliferation in gastric cancer cells.

## INTRODUCTION

Diallyl disulfide (DADS), a major oil-soluble compound derived from garlic, has multi-targeted antitumor activities in diverse cancers that result in the induction of cellular processes, including cell cycle arrest, growth inhibition, differentiation, and apoptosis, by interfering in a variety of cell signaling pathways [1–2].

We previously have reported that DADS induces gastric cancer cell differentiation by downregulating the ERK signaling pathway [3]. It induces G2/M phase cell cycle arrest by activating p38 [4], increasing histone H3 and H4 acetylation and p21^WAF1^ expression [5], and suppressing the ATR/Chk1/Cdc25C/cyclinB1 signaling pathway by specifically activating Chk1 [6–7]. Moreover, DADS inhibits the Wnt-1 signaling pathway by upregulating miR-200b and miR-22 in human gastric cancer, which leads to the apoptosis and the inhibition of proliferation [8].

DADS restrains migration and invasion in cancer cells, including the gastric cancer AGS cell line, by inhibiting the expression and activity of matrix metalloproteinases (MMPs) [9–11]. However, the mechanisms underlying its potential anti-gastric tumor metastasis activities remain to be determined. A recent study has revealed that DADS induces the reversal of the epithelial-mesenchymal transition (EMT) and inhibits growth by inactivating the β-catenin signaling pathway in breast cancer cells [12].

We have previously demonstrated that the DADS-induced downregulation of uPAR (urokinase-type plasminogen activator receptor) results in the inhibition of the ERK/Fra-1 pathway in addition to gastric cancer cell migration and invasion. DADS downregulates LIM kinase-1 (LIMK1), vimentin and MMP-9 expression and upregulates E-cadherin and TIMP-3 (tissue inhibitor of metalloproteinase-3) expression, suggesting that DADS may reverse EMT by downregulating LIMK1 [13]. In addition, we have found that the increased expression of Rac1, Pak1 and Rock1 in primary gastric cancer is correlated with lymph node metastasis, clinical stage and poor prognosis [14].

LIMK1 is involved in cell proliferation and invasion [15]. It is primarily activated by Pak, Rock and MRCK, which are downstream effectors of the Rho family of small GTPases. Among these, Rac1, Rho, and Cdc42 are considered to be major players during EMT [16]. The substrates of LIMK1 are members of the cofilin/ADF (actin-depolymerizing factor, also known as destrin) family, which maintain actin polymerization and depolymerization dynamics through their F-actin-severing activities. Inactivating cofilin/ADF by LIMK1 via phosphorylation results in the formation of membrane protrusions, which facilitates cancer cell migration and invasion [15, 17]. LIMK1 and its upstream signaling factors could be valuable targets for anti-cancer metastasis therapies [18]. DADS inhibits migration and invasion by attenuating the Rac1-Rock1/Pak1-LIMK1 pathway in colon cancer cells [19]. We therefore hypothesized that LIMK1 might be involved in DADS-induced inhibition of EMT, invasion and proliferation in gastric cancer cells.

In this study, we demonstrate that increased LIMK1 expression is associated with gastric tumor differentiation, tumor size, clinical stage, lymph node metastasis, and poor prognosis. DADS decreased Rac1, Pak1 and Rock1 expression. Overexpression and knockdown experiments performed *in vitro* revealed that DADS inhibited migration and invasion by reducing LIMK1 expression and activity. These effects may be due to the suppression of EMT, the downregulation of MMP-9 and the upregulation of TIMP-3. Downregulation of LIMK1 by DADS causes suppression of cell growth in *in vitro* and *in vivo*. Thus, DADS inhibits EMT, invasion and proliferation by downregulating LIMK1 in gastric cancer cells.

## RESULTS

### LIMK1 is upregulated in primary gastric cancer

**Figure 1:**
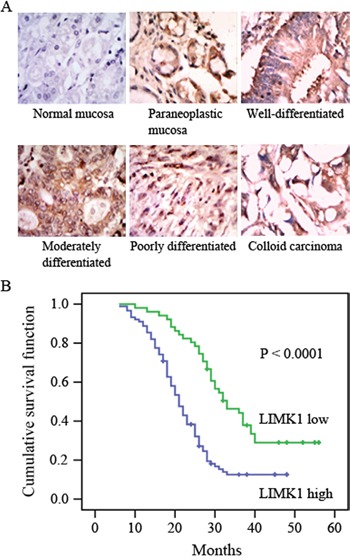
LIMK1 expression is correlated with survival probability **A.** LIMK1 expression in normal mucosa and tumor samples was detected by immunohistochemistry. A representative image is shown (×400 magnification). **B.** Increased LIMK1 expression was correlated with poor overall survival. The overall survival curves for patients with low or high LIMK1 expression are shown. These patients were right censored at the time of their last known date alive, and data until this point were used in the overall survival (OS) analysis.

To determine the relationship between LIMK1 expression and gastric cancer, LIMK1 levels were detected in 64 cases of normal stomach mucosa and 140 cases of gastric cancer tissues using IHC analysis of tissue arrays. LIMK1 expression scores greater than or less than 2 were used to divide normal and tumor tissue specimens into high-expression and low-expression groups. Out of 64 normal stomach samples, 15 (23.4%) showed high expression levels of LIMK1. In contrast, approximately 63.6% (*P* < 0.001, 89 of 140 patients) of tumors exhibited increased LIMK1 expression (Table 1). These data indicated that LIMK1 expression levels were significantly elevated in primary gastric cancer tissues compared with normal tissues. In addition, we observed that LIMK1 expression was upregulated in paraneoplastic mucosa and gastric tumors with different differentiation degrees (Figure 1A), suggesting that high LIMK1 expression levels may contribute to carcinogenesis, clinical progression and differentiation in gastric tumors.

**Table 1:** LIMK1 is upregulated in primary gastric cancer

Viable	Case (n)	Low (n)	High (n)	P-value
Normal	64	49	15	< 0.001
Gastric cancer	140	51	89	

### Increased levels of LIMK1 are correlated with tumor differentiation, invasion depth, advanced clinical stage, lymph node metastasis, and poor prognosis

The correlations between altered LIMK1 expression and clinicopathological parameters were assessed to determine potential clinicopathologic implications. As shown in Table 2, LIMK1 expression levels exhibited no significant correlation with either gender or age, but they were positively correlated with tumor differentiation (*P* = 0.001), invasion depth (*P* = 0.006), clinical stage (*P* = 0.011) and lymph node metastasis (*P* = 0.009). To further evaluate the significance of LIMK1 expression in terms of clinical prognosis, a Kaplan–Meier survival analysis was performed using patient overall survival (OS). The results showed that patients with high LIMK1 expression had fewer mean months of OS than patients with low LIMK1 expression (*P* < 0.0001 for OS, Figure 1B). Likewise, the median survival time was shorter in the high LIMK1 level group (21 months) than in the low LIMK1 level group (33 months). These results indicate that elevated LIMK1 levels are associated with tumor differentiation, tumor size, clinical stage, lymph node metastasis, andprognosis in patients with gastric cancer.

**Table 2:** Analysis of the correlation between LIMK1 expression in primary gastric cancer and its clinicopathological parameters

Viable	Case (n)	Low (n)	High (n)	P-value
Gender				
Male	97	35	62	0.898
Female	43	16	27	
Age (years)				
<60	82	31	51	0.687
≥60	58	20	38	
Histological grade				
Well differentiation	25	17	8	0.001
Moderate differentiation	37	15	22	
Poor differentiation	78	21	57	
Tumor size (cm)				
≤ 3.0	43	21	22	0.006
> 3.0	97	16	81	
TNM stage				
I-II	47	24	23	0.011
III-IV	93	27	66	
Lymph node metastasis				
Present	104	31	73	0.009
Absent	36	20	16	

### Downregulation of LIMK1 and p-LIMK1 by DADS is concomitant with the inhibition of MGC803 cell migration and invasion

**Figure 2:**
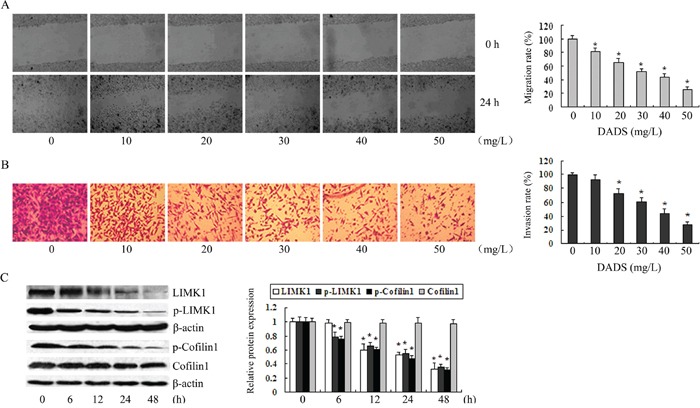
The downregulation of LIMK1 and p-LIMK1 by DADS occurred concomitantly with the inhibition of MGC803 cell migration and invasion Cells were treated with various concentrations of DADS for 24 h. **A.** Cell migration was analyzed using scratch wound assays. Migration rates are expressed as the ratio of the migration distance between treated and untreated cells. **B.** Invasion rates were determined by the ratio of the mean number of cells between treated and untreated cells. ^*^ *P* < 0.05 versus control. **C.** Western blot analysis was used to detect p-LIMK1, p-cofilin1, total LIMK1 and cofilin1 protein levels after the cells were treated with 30 mg/L DADS for the indicated times. β-actin was used as the internal control. The relative fold changes compared to controls were calculated. ^*^ *P* < 0.05 vs. control.

We first verified that DADS inhibited cell migration (Figure 2A and 2B) and decreased LIMK1 protein expression (Figure 2C) in the human gastric cancer line MGC803. These results were consistent with our previously reported data [11]. As shown in Figure 2C, p-LIMK1 levels were reduced after cells were treated with 30 mg/L DADS for 12, 24, and 48 h in a time-dependent manner, as was LIMK1 downregulation. In addition, p-cofilin1, a downstream effector of LIMK1, decreased accordingly, but there was no change in the level of total cofilin1. Intriguingly, we found that DADS downregulated p-LIMK1 and p-cofilin1 levels after 6 h incubation, which is sooner than the decrease observed in LIMK1 levels (12 h). These data indicate that DADS reduced both the total protein level of LIMK1 and its phosphorylation, which may have resulted in the decreased phosphorylation of cofilin1. The downregulation of LIMK1, p-LIMK1 and p-cofilin1 may contribute to DADS-induced inhibition of MGC803 cell migration and invasion.

### DADS downregulates Rac1, Rock1 and Pak1 expression in MGC803 cells

**Figure 3:**
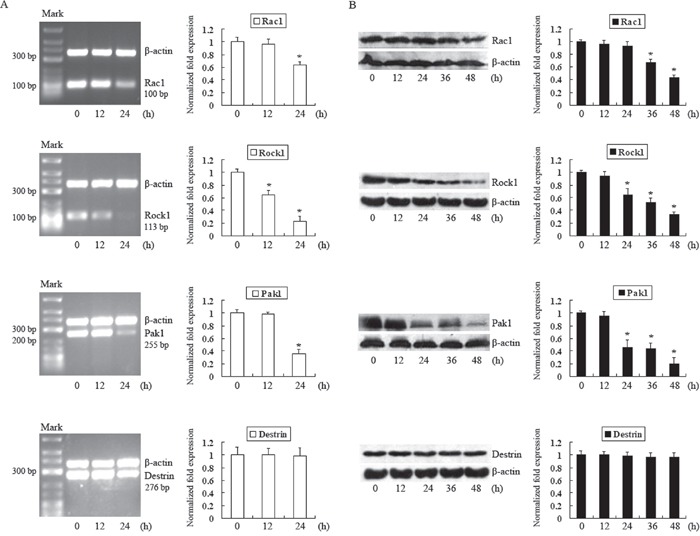
Analysis of the effects of DADS on Rac1, Rock1, Pak1 and destrin expression in MGC803 cells Cells were treated with 30 mg/L DADS for the indicated times. **A.** RT-PCR was performed to detect the mRNA levels of Rac1, Rock1, Pak1 and destrin. β-actin was used as an internal control for normalization. **B.** Western blot analysis was performed to detect the protein levels of Rac1, Rock1, Pak1 and destrin. β-actin was used as an internal control. The relative fold-changes in mRNA or protein levels compared to the controls were calculated. *P < 0.05 versus control.

We explored the effects of DADS on the expression of Rac1, Rock1 and Pak1. Cells were treated with 30 mg/L DADS for different periods of time. As shown in Figure 3, the mRNA level of Rac1 was reduced at 24 h, and Rac1 protein expression was significantly downregulated after 24 h. The level of Rock1 transcripts was decreased at 12 h, and a clear decrease in its protein level began at 24 h. The mRNA and protein levels of Pak1 were clearly reduced after 24 h of incubation. These data indicate that DADS treatment decreased the expression of Rac1, Rock1 and Pak1, which may also account for the downregulation of p-LIMK1 and p-cofilin1. In addition, we detected the expression of destrin, a downstream effector of LIMK1. No significant difference in its mRNA and protein levels were observed after DADS treatment compared to its levels in the controls. These data suggest that decreased p-LIMK1 and p-cofilin1 levels may result from the downregulation of Rac1, Rock1, Pak1 and LIMK1 expression that was induced by DADS.

### Knockdown of LIMK1 augments the inhibitory effects of DADS on MGC803 cell migration and invasion

**Figure 4:**
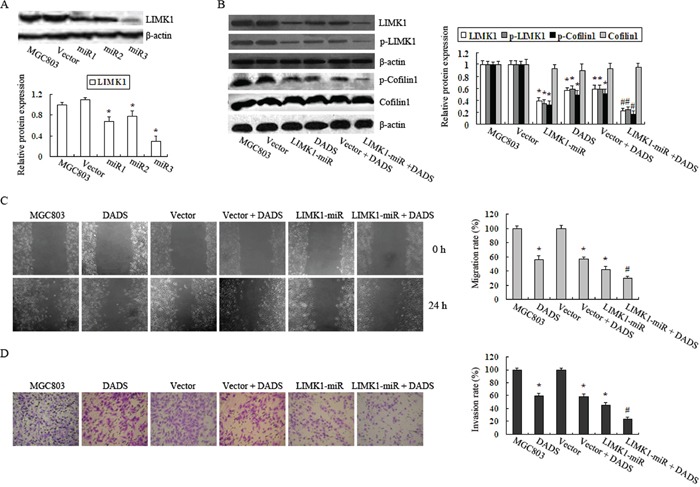
Knockdown of LIMK1 augmented the inhibitory effects of DADS on MGC803 cell migration and invasion **A.** Cells were transfected with the LIMK1-microRNA (miR1, miR2, and miR3) or empty vectors and western blot analysis was performed to detect LIMK1 protein expression levels. **B.** p-LIMK1, p-cofilin1, total LIMK1 and cofilin1 protein levels were determined by western blot analysis after the transfected or untransfected cells were treated with 30 mg/DADS for 24 h or left untreated. β-actin was used as a loading control. The relative fold-changes compared to the empty vector or the MGC803 groups were calculated. *P < 0.05 vs. MGC803 or the empty vector group. Cell migration **C.** and invasion **D.** rates compared to the MGC803 or vector groups were determined. *P < 0.05 vs. MGC803 or the vector group, ^#^P < 0.05 vs. the vector group, the vector + DADS group or the LIMK1-miR group.

To determine whether the down-regulation of LIMK1 by DADS caused the decreased activity of cofilin1 in MGC803 cells, which resulted in the inhibition of cell migration and invasion, we used microRNAs to interfere with LIMK1 protein expression. Based on the results of western blot analysis, the stable LIMK1-interfering cell line that demonstrated the best knockdown efficacy, here referred to as miR3, was chosen for the following experiments (Figure 4A). In contrast to the empty vector group, p-cofilin1, p-LIMK1 and LIMK1 levels were attenuated in the LIMK1-miRgroup (Figure 4B). Similar results were observed in cells exposed to 30 mg/L DADS for 24 h compared to untreated controls, whether we used MGC803 or the empty vector group. Moreover, DADS augmented the effects of LIMK1-miR on cells (Figure 4B). Accordingly, both the migration and the invasion rates observed in LIMK1-miR-expressing cells were significantly reduced compared to the empty vector group (Figure 4C and 4D). DADS had the same effects on MGC803 and the empty vector group. In addition, the DADS + LIMK1-miR group showed a stronger inhibitory effect than the DADS or LIMK1-miR group (Figure 4C and 4D). These data indicate that DADS inhibited cell migration and invasion by suppressing the phosphorylation of cofilin1, which resulted from suppression of LIMK1 expression and activity.

### Overexpression of LIMK1 attenuates the inhibitory effects of DADS on MGC803 cell migration and invasion

**Figure 5:**
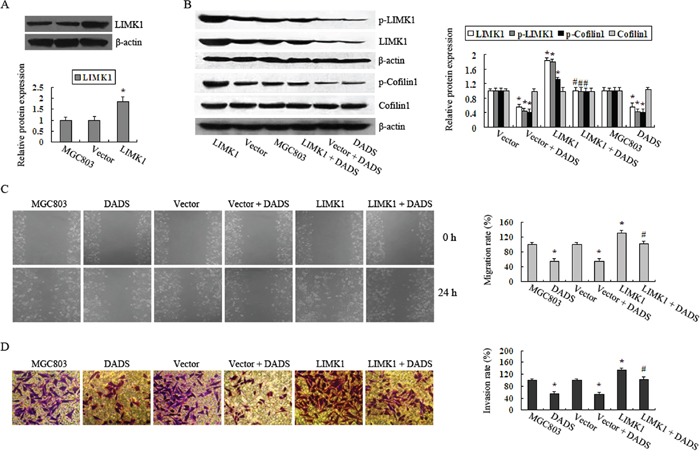
Overexpression of LIMK1 attenuated the inhibitory effects of DADS on MGC803 cell migration and invasion **A.** Cells were transfected with LIMK1-expressing or empty vectors, and western blot analysis was performed to determine the LIMK1 protein expression level. **B.** p-LIMK1, p-cofilin1, total LIMK1 and cofilin1 protein levels were determined by western blot analysis after transfected or untransfected cells were treated with 30 mg/DADS for 24 h or left untreated. β-actin was used as a loading control. The relative fold-changes compared to the empty vector group or the MGC803 group were calculated. *P < 0.05 vs. the MGC803 group or the empty vector group. Cell migration **C.** and invasion **D.** rates compared with the MGC803 group or the empty vector groups were determined. *P < 0.05 vs. the MGC803 group or the empty vector group, ^#^P < 0.05 vs. the vector + DADS group or the LIMK1 group.

To determine whether overexpressing LIMK1 has an antagonistic effect on DADS-induced inhibition of cell migration and invasion, we constructed a LIMK1-overexpressing MGC803 cell line (Figure 5A) that showed increased p-LIMK1 and p-cofilin1 levels compared to the empty vector group or the MGC803 group. After treatment with 30 mg/L DADS for 24 h, the levels of p-LIMK1 and p-cofilin1 in LIMK1-overexpressing cells were clearly reduced but were equivalent to the levels in the DADS-untreated cells (Figure 5B). In contrast, DADS-treated cells showed a stronger suppression of the phosphorylation of LIMK1 and cofilin1 in the empty vector group than in the LIMK1-overexpressing group (Figure 5B). Similarly, LIMK1 overexpression markedly increased cell activities, including migration and invasion, compared with the controls and neutralized the inhibitory effect of DADS on cell migration and invasion (Figure 5C and 5D). These results demonstrated that the overexpression of LIMK1 antagonized the DADS-induced downregulation of LIMK1 and p-LIMK1 and inhibition of cell migration and invasion.

### DADS inhibits EMT by downregulating LIMK1 in MGC803 cells

**Figure 6:**
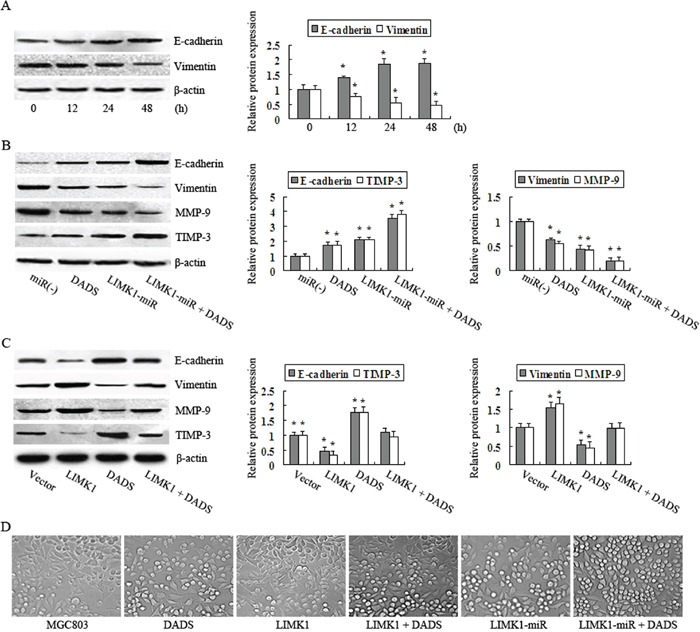
DADS inhibits EMT by downregulating LIMK1 in MGC803 cells **A.** Western blot analysis was used to detect E-cadherin and vimentin protein levels after cells were treated with 30 mg/DADS for 12, 24, and 48 h. Cells transfected with the empty, **B.** LIMK1-miR-expressing or **C.** LIMK1-expressing vectors were treated with or without 30 mg/DADS for 24 h. E-cadherin, vimentin, MMP-9 and TIMP-3 protein levels were determined by western blot analysis. β-actin was used as a loading control. The relative fold-changes compared to the empty vector groups were calculated. *P < 0.05 vs. the empty vector group. **D.** The untransfected, LIMK1-miR-expressing and LIMK1-overexpressing MGC803 cells were treated with 30 mg/DADS for 24 h or left untreated. Representative images were captured by phase-contrast microscopy.

Vimentin and E-cadherin protein levels were decreased and increased, respectively, by 30 mg/L DADS in a time-dependent manner (Figure 6A). These data suggest that DADS inhibits EMT in gastric cancer cells, which may explain its suppressive effects on MGC803 cell migration and invasion. To further determine whether LIMK1 is involved in the DADS-triggered inhibition of EMT in MGC803 cells, we explored the effects of both the knockdown and overexpression of LIMK1 on EMT in DADS-treated and -untreated cells. LIMK1 knockdown led to the upregulation of E-cadherin and TIMP-3 and the downregulation of vimentin and MMP-9. These effects were similar to those observed in cells treated with DADS alone, and they were enhanced in DADS-treated cells compared with untreated MGC803 cells (Figure 6B). LIMK1 overexpression therefore neutralized the effects of DADS (Figure 6C).

We then assessed changes in cell morphology. In the MGC803 group, many cells had a typical spindle-shape morphology and demonstrated a reduction in cell-cell junctions. In contrast, many cells grew in clusters with well-defined junctions and exhibited an epithelial-like morphology in the DADS-treated group and the LIMK1-miR group. However, the majority of cells in the LIMK1-overexpressing group demonstrated mesenchymal cell morphology and almost no cell-cell junctions. In addition, LIMK1 overexpression antagonized the DADS-induced changes in cell morphology. These data collectively indicate that LIMK1 is required for EMT in MGC803 cells and that the downregulation of LIMK1 by DADS slowed EMT.

### Effects of LIMK1 knockdown and overexpression on DADS-induced growth inhibition in MGC803 cells *in vitro*

**Figure 7:**
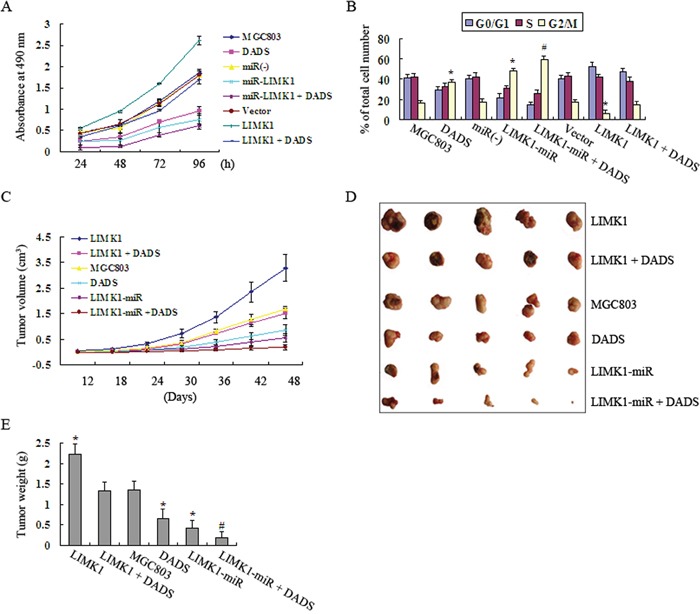
Effects of LIMK1 knockdown and overexpression on the DADS-induced inhibition of growth in MGC803 cells *in vitro* and *in vivo* **A.** Cell proliferation assays were performed *in vitro* after the untransfected, LIMK1-miR-expressing and LIMK1-overexpressing MGC803 cells were treated with 30 mg/DADS or left untreated for the indicated times. **B.** Cells were treated with 30 mg/L DADS for 24 h or left untreated. The percentages of cells in the G0/G1, S and G2/M phases of the cell cycle were determined by flow cytometry. **C.** Untransfected, LIMK1-miR-expressing and LIMK1-overexpressing MGC803 cells were injected into the subcutis of nude mice. The mice were treated with normal saline or DADS via intraperitoneal injection every 2 days. The effect of DADS, LIMK1 knockdown and LIMK1 overexpression on tumor volume was examined every 6 days. Average tumor volumes are represented (n=5 per group) starting from the twelfth day and continuing until sacrifice at 48 days. **D.** The xenografts were collected at 48 days. Tumor sizes are shown for each group of MGC803 model mice. **E.** The mean ± SD tumor weight for each group was calculated at the termination of the experiment. *P < 0.05 vs. the MGC803 group, vector group, or the LIMK1 + DADS group. ^#^P < 0.05 vs. the MGC803 group, DADS group, miR(−) group, or LIMK1-miR group.

We performed *in vitro* experiments to explore the biological effect of LIMK1 on MGC803 cell growth and to determine whether there is an association between DADS antiproliferative effects and LIMK1 expression. Cell proliferation was assessed *in vitro* after untransfected, LIMK1-miR-expressing and LIMK1-overexpressing MGC803 cells were treated with 30 mg/DADS or left untreated for different periods of time. As shown in Figure 7A, the empty vector groups showed no significant change in cell proliferation compared to that of the MGC803 group. Cell proliferation was decreased in the DADS and LIMK1 knockdown groups compared to the control groups. The effects of DADS were augmented by knockdown of LIMK1, whereas these effects were attenuated by overexpression of LIMK1. As shown in Figure 7B, flow cytometry analysis revealed that 37.1% of the cells were arrested in G2/M phase in the DADS groups compared to 16.4% in the MGC803 group. In the LIMK1 knockdown group, the percentage of G2/M phase cells was increased to 47.6% compared to 17.3% in the negative control group. Moreover, the proportion of G2/M cells was increased by DADS treatment in the LIMK1-miR + DADS group (59.3%). In contrast, LIMK1 overexpression reduced the proportion of G2/M cells (6.1%) compared to the empty vector group (16.8%). DADS arrested 14.3% cells in G2/M phase in the LIMK1 + DADS group.

### Effects of LIMK1 knockdown and overexpression on DADS-induced growth inhibition in MGC803 cells *in vivo*

For these *in vivo* experiments, MGC803 cells were subcutaneously injected into nude mice, and the mice were subjected to different treatments, as shown in the materials and methods section. Tumor volume was examined every 6 days. According to Figure 7C, compared to the MGC803 control group, the DADS and LIMK1-miR groups exhibited a detectable reduction in tumor volume, whereas an increased tumor volume was observed in the LIMK1-overexpressing group. Furthermore, mice treated with LIMK1 + DADS or LIMK1-miR exhibited reduced or increased tumor volumes, respectively, in contrast to those treated with DADS alone. After 48 days, the xenografts were removed. The same changes were observed in tumor volume and weight (Figure 7D and 7E). These *in vitro* and *in vivo* data indicate that LIMK1 may promote the G2/M transition in MGC803 cells, and the downregulation of LIMK1 could therefore explain DADS-induced cell cycle arrest in G2/M phase, which results in the inhibition of cell proliferation.

### Effects of LIMK1 knockdown and overexpression on DADS-induced vimentin, CD34, Ki-67 and E-cadherin expression *in vivo*

**Figure 8:**
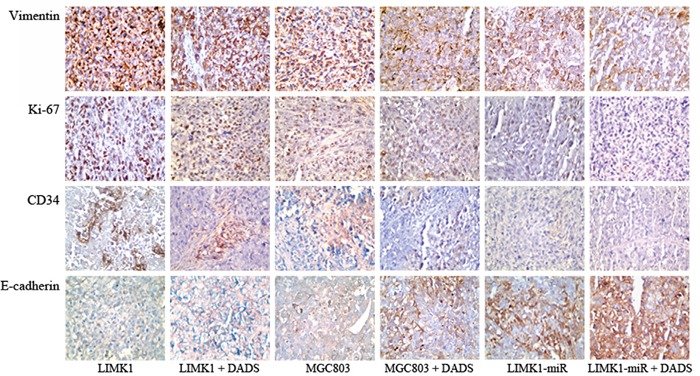
Effects of LIMK1 knockdown and LIMK1 overexpression on DADS-induced vimentin, CD34, Ki-67 and E-cadherin expression *in vivo* Untransfected, LIMK1-miR-expressing and LIMK1-overexpressing MGC803 cells were injected into the subcutis of nude mice. The mice were treated with normal saline or DADS via intraperitoneal injection every 2 days. The xenografts were collected at 48 days. Immunohistochemistry was performed to detect the expression of vimentin, CD34, Ki-67 and E-cadherin in the tumor tissue specimens obtained from the xenografts. A representative tissue section is shown for each group (×400 magnification).

Immunohistochemistry was used to detect the protein expression levels of vimentin, CD34, Ki-67 and E-cadherin in transplanted tumor tissues. Vimentin and CD34 are associated with tumor growth, invasion and metastasis, and Ki-67 is a marker of tumor cell proliferation. As shown in Figure 8, vimentin, CD34, and Ki-67 expression levels were downregulated by DADS and LIMK1-miR. LIMK1-miR potentiated the inhibitory effect of DADS on the expression of these markers. LIMK1 overexpression, which increased their expression, attenuated the inhibitory effects of DADS on their expression. The downregulation of E-cadherin has been demonstrated to facilitate tumor cell invasion and metastasis. Its expression was increased by DADS and LIMK1-miR and decreased by LIMK1 overexpression. In contrast to the effects of treatment with DADS alone, the effect of DADS on E-cadherin expression was enhanced by LIMK1-miR and antagonized by LIMK1 overexpression (Figure 8).

## DISCUSSION

In this study, we first confirmed that LIMK1 is upregulated in primary gastric cancer and that a higher level of LIMK1 expression is correlated with tumor differentiation, tumor size, advanced clinical stage, lymph node metastasis, and poor prognosis, suggesting that LIMK1 may contribute to carcinogenesis and the clinical progression of gastric cancer. We next explored the effects of DADS on the Rac1-Pak1/Rock1-LIMK1 signaling pathway, focusing on whether the downregulation of LIMK1 is associated with DADS-induced inhibition of EMT, invasion and proliferation.

The inhibition of either the expression or the activity of Rac1 [20–21], Pak1 [22–23] or Rock1 [24–25] leads to the suppression of tumor cell growth, invasion and metastasis. We found that the expression levels of Rac1, Pak1, and Rock1 were reduced by treatment with DADS in MGC803 cells. Recent studies have shown that inhibiting Rac1 activity [26] or knocking down LIMK1 expression [27] abrogated their effects on malignant phenotypes, including increased growth and invasion in gastric cancer cells. Thus, the downregulation of the Rac1-Pak1/Rock1 pathway by DADS may result in the suppression of LIMK1 activation and thereby decrease cell growth and invasion.

LIMK is required for the formation of invadapodia, which are involved in MMP-mediated extracellular matrix degradation during tumor cell invasion [17]. LIMK is primarily engaged in modulating cofilin/ADF activity [28–29]. Cofilin/ADF and LIMK1 are concurrently overexpressed and overactivated in invading cells *in vitro* and in metastatic cells *in vivo* in diverse tumors that are associated with invasion, intravasation and metastasis [30–31]. Although we observed no alteration in cofilin1 and destrin expression levels, the downregulation of p-cofilin1 by DADS occurred concomitantly with a decrease in LIMK1 and p-LIMK1 levels, and these changes were consistent with the reduced rates of cell migration and invasion.

Interestingly, we found that a decrease in p-LIMK1 occurred earlier than the decreases in LIMK1 and its upstream modulators (Rac1, Pak1, and Rock1) after cells were incubated with DADS. This indicates that the reduced levels of p-LIMK1 may be the result of DADS inhibiting its activation via other mechanisms in addition to decreasing Rac1, Pak1, Rock1 and LIMK1 expression.

Inactivation of cofilin by LIMK1 is required for local F-actin stability and the formation and maturation of functional invadopodia [17]. Because knockdown of LIMK1 or cofilin1 expression decreases cancer cell motility and invasion [16, 31, 32], we proposed that cofilin phosphorylation resulting from LIMK1 overexpression and overactivation may lead to cell migration and invasion in gastric cancer cells. This assumption was verified by the results of *in vitro* experiments that DADS inhibited cofilin phosphorylation and that cell migration and invasion were attenuated and augmented by overexpression and knockdown of LIMK1, respectively. Thus, DADS suppresses the LIMK1-cofilin1 pathway, resulting in the DADS-induced inhibition of migration and invasion in gastric cancer cells.

The Rac1-Pak1-LIMK1-cofilin1 pathway is involved in EMT [33–35]. We have previously demonstrated that DADS increased E-cadherin and TIMP-3 (an inhibitor of MMPs) expression and decreased vimentin and MMP-9 expression in MGC803 cells *in vitro*. We therefore have proposed that DADS may suppress migration and invasion by inhibiting EMT [13]. In this study, we verified that DADS and LIMK1 knockdown induced changes in cell morphology *in vitro*, and we showed that this had the same effects on the expression of vimentin, E-cadherin, MMP-9 and TIMP-3. The changes in the expression of these proteins that were induced by DADS were antagonized by LIMK1 overexpression. Furthermore, the effects of LIMK1 overexpression and knockdown on DADS-induced vimentin and E-cadherin expression were supported by the results from *in vivo* experiments. Therefore, LIMK1 promotes EMT in gastric cancer cells, and this process can be reversed by DADS.

High vimentin expression and low E-cadherin expression are observed in various epithelial cancers, including gastric cancer, and are correlated with EMT, tumor growth, invasion, and poor prognosis [36–37]. Overexpression of LIMK1 accelerates the activation of ERK [15], which increases vimentin [38] and decreases E-cadherin [39], and the LIMK1/cofilin1 pathway is involved in EMT [35]. We reported that DADS inhibited the ERK/Fra-1 pathway, increased E-cadherin expression and decreased vimentin expression in MGC803 cells *in vitro* [13]. Thus, the effects of DADS on E-cadherin and vimentin expression may result from the blocking of the ERK/Fra-1 pathway by the inhibition of LIMK1 expression and activity.

In *in vitro* experiments, we observed that LIMK1 overexpression promoted the G2/M transition and cell proliferation and blocked the DADS-induced inhibition of proliferation, whereas knockdown of LIMK1 exhibited effects similar to those of DADS. Moreover, the effect of LIMK1 knockdown and overexpression on the DADS-induced inhibition of tumor growth were confirmed in *in vivo* experiments. LIMK knockdown causes cells to accumulate in G2/M phase [40–41]. Thus, these results reveal that the downregulation of LIMK1 by DADS results in G2/M phase arrest and therefore the inhibition of cell proliferation.

Ki-67 [42] and CD34 [43] are markers of cell proliferation and angiogenesis, respectively, in tumors. Pak1, an upstream activator of LIMK1, is associated with the expression of CD34 [43] and Ki-67 [44]. We demonstrated that overexpression of LIMK1 increased Ki-67 and CD34 expression in xenografted tumors. DADS treatment and the knockdown of LIMK1 expression had opposite effects. Therefore, the downregulation of LIMK1 may result in DADS-induced changes in the expression of these proteins and inhibit proliferation in gastric cancer.

In summary, we confirmed that DADS reduces the expression of Rac1, Pak1 and Rock1 in gastric cancer MGC803 cells. Moreover, DADS inhibits EMT, invasion and proliferation by downregulating LIMK1. These effects are associated with the decreased expression of vimentin, MMP-9, CD34, and Ki-67 and the increased expression of E-cadherin and TIMP-3. Therefore, downregulation of LIMK1 may explain, in part, the mechanisms by which DADS inhibits EMT, invasion and proliferation in gastric tumor cells.

## MATERIALS AND METHODS

### Reagents and antibodies

DADS, purchased from Fluka Co. (Milwaukee, Wisconsin, USA), was dissolved in Tween-80 and stored at −20°C after a 100-fold dilution with saline. The primary antibodies against cofilin1 (M1), p-cofilin1 (S3), LIMK1 (Q491) and p-LIMK1 (T508), Horseradish peroxidase (HRP)-conjugated secondary antibodies were provided by Abzoom (Dallas, TX, USA). The primary antibodies against Pak1 and Rock1 were purchased from Epitomics (Burlingame, CA, USA). The primary antibodies against destrin (ab11072), E-cadherin (ab40772), vimentin (ab92547), Ki-67 (ab66155), CD34 (ab81289), MMP-9 (ab38898) and TIMP-3 (ab39184) were provided by Abcam (Cambridge, MA, UK). Mouse monoclonal antibody against Rac1 (23A8) was purchased from Millipore (Billerica, MA, USA). The mouse monoclonal against β-actin antibody (sc-8432) was purchased from Santa Cruz Biotechnology (Santa Cruz, CA, USA).

### Clinical samples

All tissue samples used in the present study were collected from the First Affiliated Hospital, University of South China (Hengyang, Hunan, China). Written informed consent was obtained from each patient. The collection and use of tissues followed the procedures according to the ethical standards as formulated in the Helsinki Declaration. This study was approved by the research ethics committee of University of South China.

The tissue microarrays, including 140 cases of gastric cancer and 64 cases of normal stomach mucosa, were used for immunohistochemistry (IHC) analysis. All data, including age, sex, histologic grade, tumor size, and lymph node metastasis were obtained from clinical and pathologic records.

### Analysis of LIMK1 expression in gastric cancer

IHC was performed to detect LIMK1 expression in normal stomach mucosa and gastric cancer. The expression levels of LIMK1 were evaluated independently by three pathologists who were blinded to the patient's clinical data. The intensities of positive staining were scored by 0-4, according to the standards of 0-1 (no staining), 1-2 (weak staining), 2-3 (medium staining), and 3-4 (strong staining). The percentages of positive stained cells were analyzed. Those expression scores equaled to scores of the intensities × the percentages of positive cells. Those expression scores of ≥ 2 was defined as high expression, < 2 was considered as low expression.

### Cell culture and cell line establishment

Human gastric cancer MGC803 cell line was obtained from the Cancer Research Institute, Xiangya Medical College, Central South University in China. Cells were cultured in RPMI-1640 medium containing 10% fetal bovine serum (Gibco, Life Technologies, Vienna, Austria) with the addition of 100 U/mL penicillin, 100 U/mL streptomycin, and maintained at 37°C in a humidified atmosphere of containing 5% CO_2_. To establish the stable LIMK1-interfering cell lines, three pcDNA™6.2-GW/EmGFPmiR LIMK1-microRNA (miR)-expressing plasmids were constructed by Invitrogen Corporation. Sequences of DNA oligomers inserted into pcDNA™6.2-GW/EmGFPmiR were listed as follow, miR1: sense, 5′-TGCTGATGGAGTGGAGGTAGGCCATCGTTTTGGC CACTGACTGACGATGGCCTCTCCACTCCAT-3′ and antisense, 5′-CCTGATGGAGTGGAGAGGCCATC GTCAGTCAGTGGCCAAAACGATGGCCTACCTCCA CTCCATC-3′; miR2: sense, 5′-TGCTGACCTGAAGCA GTCTGCGTGCCGTTTTGGCCACTGACTGACGGCA CGCACTGCTTCAGGT-3′ and antisense, 5′-CCTGAC CTGAAGCAGTGCGTGCCGTCAGTCAGTGGCCAAA ACGGCACGCAGACTGCTTCAGGTC-3′; miR3: sense, 5′-TGCTGACGTGAGGCAGATGAAACACTGTTTT GGCCACTGACTGACAGTGTTTCCTGCCTCACGT-3′ and antisense, 5′-CCTGACGTGAGGCAGGAAACA CTGTCAGTCAGTGGCCAAAACAGTGTTTCATCTG CCTCACGTC-3′; MGC803 cells were transfected with LIMK1-miR expressing plasmid and empty vector (control) using Lipofectamine 2000 reagent (Invitrogen, Carlsbad, California, USA), following the manufacturer's instructions. The transfected cells were selected with blasticidin (Invitrogen). The expression levels of LIMK1 were evaluated by Western blotting analysis to confirm the knockdown efficacy. To establish LIMK1-overexpressing cell lines, MGC803 cells were transfected with pIRES2-EGFP LIMK1-expressing plasmid (constructed by Invitrogen Corporation) and empty vector (control). The transfected cells were selected with G418 (Invitrogen) until the stable transgene expression during culture maintenance. The LIMK1 expression levels in stable cell lines were evaluated by Western blot analysis.

### Reverse transcription-polymerase chain reaction (RT-PCR)

After cells were untreated or treated with DADS (Fluka Co, Milwaukee, Wisconsin, USA), total RNA was extracted from the cells using Trizol reagent (Gibco BRL, Grand Island, USA). Reverse transcription was carried out using the RT-PCR system (Promega, Madison, USA). PCR analysis was performed using the Gene amp PCR kit (Promega). Primer sequence for Rac1 forward: CCCTATCCTATCCGCAAACA, reverse: CGC ACC TCA GGA TAC CAC TT; Rock1 forward: AAA ACC TTA TTT GTG CCT TCC, reverse: CGT TTC CCA AGC CCA CT; Pak1 forward: AAG ACA TCC AAC AGC CAG AA, reverse: TGT AGC CAC GTC CCG AGT; Destrin forward: TGG TTG GAG ATG TTG GTG, reverse: TAC AAG CCC GAT TGA GAT; β-actin forward: ACA CTG TGC CCA TCT ACG AGG GG, reverse: ATG ATG GAG TTG AAG GTA GTT TCG TGG AT. The PCR products were analyzed on 2% agarose gel containing ethidium bromide. Densito-metric quantitation of products was determined using the Labwork analysis software. The relative abundance was expressed as the ratio of the object gene to β-actin.

### Western blot analysis

For total protein extraction, cells lysed directly on ice for 30 min in lysis buffer [10 mmol/L Tris-HCl (pH 7.6), 100 mmol/L NaCl, 1 mmol/L EDTA (pH 8.0), 100 μg/mL PMSF and 1 μg/mL aprotinin]. The cell lysates were centrifugated at 12,000 rpm for 10 min and the supernatants were collected. Then protein contents were determined using a BCA protein assay kit (Pierce, Rockford, IL, USA).

Protein extracts were loaded on a 10% SDS-polyacrylamide gel for electrophoresis and transferred onto Polyvinylidene Fluoride (PVDF) membrane. The blots were blocked in 5% skim milk in Tris buffered saline (TBS) containing 0.1% Tween 20 for 2 h at room temperature, and then incubated with the primary antibody at 4°C overnight. The membranes were washed in TBS-T and then incubated with horseradish peroxidase (HRP)-conjugated secondary antibody (1:1000-1:2000). After washed with TBS-T, the membranes were developed by an enhanced chemiluminescence plus (ECL Plus) kit (Amersham Biosciences, Buckinghamshire, England) and bands were visualized on X-ray film (Kodak). Membranes were re-incubated with anti-β-actin antibody to verify equal protein-sample loading. The target protein amounts were normalized towards β-actin quantity using densitometry, then relative fold changes in protein levels were calculated as ratios between treated versus control group values.

### Cell migration and invasion assays

Cell migration and invasion assays were performed as previously described [13]. For the cell migration assays, an artificial “wound” was created after transfected and untransfected MGC803 cells were cultured to 90% confluence. Cells were left untreated or were treated with DADS at the indicated concentrations for 24 h, and the wound areas were then photographed using an inverted microscope. The migration distance was measured, and migration rates are expressed as the ratio of the treated group value to the control group value. Invasion assays were performed using Transwell^®^ plates (Corning, Corning, NY). Briefly, MGC803 cells were seeded onto Matrigel-coated filters. Transfected and untransfected cells were treated with indicated concentrations of DADS for 24 h or left untreated. The cells that had invaded the lower surface of the filter were fixed and stained with hematoxylin. Invasion rates are expressed as the ratio of the treated group value to the control group value.

### Cell proliferation assays and cell cycle analysis

Cell proliferation assays were performed using a CellTiter 96 AQ One Solution Cell Proliferation Assay kit (MTS, Promega, Madison, USA) according to the manufacturer's instructions. Cells were seeded in 96-well plates at 1 × 10^4^ cells per well. Transfected and untransfected cells were treated with 30 mg/L DADS for the indicated times or left untreated, and the absorbance was recorded at 490 nm using an ELISA plate reader. Each assay was replicated 5 times.

Cell cycle analyses were conducted as follows. Briefly, transfected and untransfected cells were treated with 30 mg/L DADS for 24 h or left untreated. Cells were harvested and resuspended in ice-cold 75% ethanol and then fixed for 24 h at 4°C. For subsequent flow cytometry analysis, fixed cells were resuspended in 1 mL of PI (propidium iodide) staining reagent for 30 min. The data were collected using a FACScan flow cytometer (Becton Dickinson, Franklin Lakes, NJ, USA) and analyzed with Verity Winlist Software (Verity Software House, Topsham, ME, USA).

### Gastric tumor growth in nude mice

Untransfected or transfected MGC803 cells were injected into the subcutis of the right axillary of male athymic BALB/c nude mice (4 weeks old). The mice were treated with normal saline or 100 mg/kg DADS via intraperitoneal injection every 2 days until the termination of the experiment. Tumor volume (cm^3^) was examined every 6 days and calculated using a standard formula (width^2^ × length × 0.5). Average tumor volumes are presented (n=5 for each group) starting from the twelfth day and continuing until sacrifice at 48 days. The xenografts were removed, and tumor size and weight were measured at 48 days. Tumor tissues were then fixed in formalin and embedded in paraffin. Tissue sections (5 μm-thick) were prepared for subsequent IHC analysis. All experiments were performed according to the guidelines for animal use of the Ethics Committee of University of South China.

### Immunohistochemistry

Briefly, after slides were dewaxed in xylene and hydrated in graded alcohol solutions, antigen retrieval was performed by heat treatment in 10 mM sodium citrate buffer (pH 8.0). Slides were incubated in 3% H_2_O_2_ solution to quench endogenous peroxidase activity and then incubated with normal goat serum for 20 min. Slides were incubated with primary antibodies (dilution 1:100) at 4°C overnight. Positive signals were developed with peroxidase-conjugated secondary antibodies and 0.5% diaminobenzidine/H_2_O_2_ followed by counterstaining with Mayer's hematoxylin, dehydration, clearing, and mounting. The slides that were treated with normal goat serum were evaluated as negative controls.

### Statistical analysis

All results are presented as the mean ± SD for three independent experiments. Student's *t* tests and one-way ANOVA were used to analyze differences in expression among groups.

Pearson's χ^2^ test was used to analyze differences in LIMK1 expression between normal gastric epithelia and tumor samples and to evaluate correlations between LIMK1 expression and clinicopathological parameters. Univariate survival analysis was conducted according to the Kaplan–Meier method, with log-rank tests for comparison. Survival was measured from the day of the surgery. *P*-values < 0.05 were considered to be statistically significant. Statistical analyses were conducted using SPSS13.0 software.
